# A modified oral sugar test for evaluation of insulin and glucose dynamics in horses

**DOI:** 10.1186/1751-0147-57-S1-O4

**Published:** 2015-09-25

**Authors:** Sanna Lindåse, Katarina Nostell, Ida Askerfelt, Johan Bröjer

**Affiliations:** 1 Department of Clinical Sciences, Swedish University of Agricultural Sciences, Uppsala, Sweden

## Introduction

An oral sugar test (OST) using Karo® Light Corn Syrup has been developed in the USA as a field test for the assessment of insulin sensitivity in horses but the syrup is not available in Europe.

## Objectives

The aim of the study was to compare the results of a modified OST between horses with equine metabolic syndrome (EMS) and healthy horses using a Scandinavian commercially available glucose syrup (Dansukker glykossirap). In addition, the effect of breed and the repeatability of the test were evaluated.

## Methods

Clinically healthy horses of different breeds (7 Shetland ponies, 8 Icelandic horses, 8 Standardbred horses) and 15 horses with EMS were included. The Icelandic horses and Shetland ponies underwent the OST twice. Insulin and glucose data from the OST were used to calculate several parameters e.g. peak insulin concentration (PI), area under the curve for insulin (AUCins) and insulin sensitivity index by Matsuda (ISI-Matsuda).

## Results

There was no effect of breed in the group of healthy horses on PI, AUCins and ISI-Matsuda. The EMS horses had 5 - 7 times as high means for PI, AUCins and ISI-Matsuda as the clinically healthy horses. Coefficient of variation for repeated tests was 19.8 %, 19.0 % and 17.6 % for PI, AUCins and ISI-Matsuda respectively.

## Conclusions

The modified OST appears to be a useful field screening test to determine whether the horse is IR or not. Estimates derived from the OST may also be useful to estimate insulin sensitivity but requires further evaluation.

